# Capturing pregnancy recognition trajectories: a critical reflection of new quantitative measures tested in Ethiopia, Malawi, Nigeria and Zambia

**DOI:** 10.1080/26410397.2025.2531684

**Published:** 2025-08-12

**Authors:** Joe Strong, Ann M. Moore, Ernestina Coast, Onikepe Owolabi, Tamara Fetters

**Affiliations:** a Visiting Fellow, Department of International Development, London School of Economics and Political Science, London, UK; b Principal Research Scientist, Guttmacher Institute, New York, NY, USA; c Professor of Health and International Development, Department of International Development, London School of Economics and Political Science, London, UK.; d Vice President for International Research, Guttmacher Institute, New York, NY, USA; e Senior Research Scientist, IPAS, Chapel Hill, NC, USA

**Keywords:** pregnancy, pregnancy recognition, abortion, Ethiopia, Malawi, Nigeria, Zambia

## Abstract

The experiences and timings of pregnancy recognition trajectories have significant impacts on pregnancy-related care. Understanding individuals’ contextually informed trajectories is crucial to their reproductive rights and service delivery needs. While many studies take pregnancy recognition as a starting point, capturing the complexities and nuances of these trajectories has received much less attention. This paper critically reflects on new approaches to capturing pregnancy recognition trajectories in two studies conducted between 2018 and 19, one in Nigeria with women aged 18 and over (*n* = 394), and a three-country study with adolescents aged 10–19 in Ethiopia, Malawi, and Zambia (*n* = 313). Pregnancy recognition trajectories were complex and involved multiple physical, material, and psychological recognition factors. Adolescents in the three-country study cited predominantly between two and four factors that led to their pregnancy recognition, with a range of one to seven factors. In the Nigerian study, 43.4% of respondents named two factors that led them to recognise they were pregnant, with a range of one to five factors. As pregnancy recognition is the starting point for many public health actions and interventions, it is imperative that future survey tools better capture this complex and poorly understood process. Our analyses suggest that questions should include response categories that capture physical, material, and psychological contributors to pregnancy recognition, including open-ended responses to capture heretofore unidentified aspects of this process. Questions on the duration of time between recognition factors would be beneficial, as well as an understanding of what factors were most important to an individual when recognising a pregnancy.

## Plain language summary

The ways in which people come to recognise they are pregnant can be complex, nuanced, and varied. These trajectories can have serious implications for their pregnancy experiences, including access to different types of pregnancy-related care. That could include antenatal care and abortion care. Currently, there are few studies that examine these trajectories. Thus, there is a large evidence gap around pregnancy recognition. New tools for capturing the complexities of pregnancy recognition are needed to fill this evidence gap. This paper draws on two studies that used different and new approaches to understand pregnancy recognition. One study conducted research with women in Nigeria aged 18 and over, the other with adolescents aged 10–19 in Ethiopia, Malawi, and Zambia. It was important that questions allowed participants to describe various ways that they recognised their pregnancy. Women and girls in both studies reported having several different reasons to think they might be pregnant. That could include taking multiple medical pregnancy tests. Questions that can capture the physical, material, and psychological contributors to pregnancy recognition are important. Other key people (such as parents, partners, or medical providers) are also significant in recognition. It is also important to understand how long it took someone to recognise and put together evidence of a pregnancy. This needs to be captured in future research.

## Résumé

Les expériences et le choix des moments des trajectoires de reconnaissance de la grossesse ont des conséquences importantes sur les soins en lien avec la grossesse. Il est essentiel de comprendre les trajectoires individuelles, éclairées par le contexte, pour les droits reproductifs des personnes et leurs besoins en matière de prestation de services. Si de nombreuses études prennent la reconnaissance de la grossesse comme point de départ, elles sont beaucoup plus rares à tenter de saisir les complexités et les nuances de ces trajectoires. Cet article examine de manière critique les nouvelles approches pour saisir les trajectoires de reconnaissance de la grossesse dans deux études réalisées entre 2018 et 2019, l’une au Nigéria auprès de femmes âgées de 18 ans et plus (*n* = 394) et l’autre dans trois pays auprès d’adolescentes âgées de 10 à 19 ans en Éthiopie, au Malawi et en Zambie (*n* = 313). Les trajectoires de reconnaissance de la grossesse étaient complexes et comportaient de multiples facteurs de reconnaissance physiques, matériels et psychologiques. Les adolescentes de l’étude menée dans les trois pays ont cité principalement de deux à quatre facteurs ayant conduit reconnaître qu’elles étaient enceintes, avec une fourchette d’un à sept facteurs. Dans l’étude nigériane, 43,4% des répondantes ont cité deux facteurs qui les ont amenées à reconnaître leur grossesse, avec une fourchette d’un à cinq facteurs. Étant donné que la reconnaissance de la grossesse est le point de départ de beaucoup d’activités et d’interventions de santé publique, il est impératif que les futurs outils d’enquête rendent mieux compte de ce processus complexe et mal compris. Notre analyse propose que les questions incluent des catégories de réponses qui prennent en compte les facteurs physiques, matériels et psychologiques de la reconnaissance de la grossesse, notamment des réponses ouvertes pour saisir des aspects jusqu’ici non identifiés de ce processus. Des questions sur le délai entre les facteurs de reconnaissance seraient utiles, ainsi qu’une meilleure compréhension des facteurs les plus importants pour une personne lors de la reconnaissance d’une grossesse.

## Resumen

Las experiencias y los momentos en que ocurren las trayectorias de reconocimiento del embarazo tienen impactos significativos en la atención relacionada con el embarazo. Entender las trayectorias informadas contextualmente de las personas es fundamental para sus derechos reproductivos y sus necesidades de prestación de servicios. Si bien muchos estudios consideran el reconocimiento del embarazo como punto de partida, la captura de las complejidades y matices de estas trayectorias ha recibido mucho menos atención. Este artículo reflexiona críticamente sobre nuevos enfoques para capturar las trayectorias de reconocimiento del embarazo en dos estudios realizados entre 2018 y 2019, uno en Nigeria con mujeres de 18 años o más (*n* = 394) y otro con adolescentes entre 10 y 19 años realizado en tres países: Etiopía, Malaui y Zambia (*n* = 313). Las trayectorias de reconocimiento del embarazo eran complejas e implicaban varios factores físicos, materiales y psicológicos. Las adolescentes en el estudio realizado en tres países citaron predominantemente entre dos y cuatro factores que llevaron a su reconocimiento del embarazo, en una gama de uno a siete factores. En el estudio en Nigeria, 43.4% de las encuestadas mencionaron dos factores que las llevaron a reconocer que estaban embarazadas, en una gama de uno a cinco factores. Dado que el reconocimiento del embarazo es el punto de partida para muchas acciones e intervenciones de salud pública, es imperativo que futuras herramientas de encuestas capturen mejor este proceso complejo y mal entendido. Nuestros análisis indican que las preguntas deben incluir categorías de respuestas que capturen los contribuidores físicos, materiales y psicológicos al reconocimiento del embarazo, incluidas las respuestas abiertas para capturar aspectos hasta ahora no identificados de este proceso. Sería beneficioso tener preguntas sobre la duración de tiempo entre factores de reconocimiento, así como una comprensión de los factores más importantes para cada persona para reconocer un embarazo.

## Introduction

Pregnancy recognition trajectories are the processes by which a person comes to recognise that they are pregnant (Strong, Coast et al. 2023). Pregnancy recognition incorporates a number of intersecting and non-linear experiences that might include ignoring, suspecting, denying, rejecting, confirming, and comprehending the presence of a pregnancy (Strong, Coast et al. 2023). How, when, and in what ways someone comes to recognise they are pregnant can have significant implications for their pregnancy-related care, pregnancy outcomes, and their sexual and reproductive rights, ranging from access to antenatal care to abortion decision-making.^1–4^ Thus, it is essential for sexual and reproductive health researchers, providers, and policymakers to better understand pregnancy recognition trajectories.

Trajectories can be complex and influenced by factors, including age, health literacy, co-morbidities, intentions and expectations of getting pregnant, perceptions of exposure to pregnancy risk, access to pregnancy recognition technologies, cultural health capital, and more^5,6^. These factors may be tied to socio-economic and socio-demographic factors that can shape pregnancy recognition, such as previous experiences of pregnancy, marital status, or financial resources^1,6–8^ A common sign of pregnancy, a missed period, can be complicated when the individual has had prior experiences of irregular menstruation, continued bleeding throughout a pregnancy, and/or has paid limited attention to their menstrual cycles.^9–11^

Bodily changes and symptoms such as nausea and fatigue can be associated with conditions other than pregnancy.^4,12,13,14^ Beliefs around the risk of a pregnancy occurring can also impact recognition trajectories, which are influenced by an individual’s knowledge of how and when pregnancies occur, whether they are fecund, and whether they believe that they are protected e.g. through contraceptive use.^13,14^ Trajectories may also be shaped by access to and use of pregnancy recognition technologies (e.g. medical tests), as well as interactions with healthcare providers.^15,16^ Psychological factors may delay recognition, including denial of signs and symptoms of pregnancy and/or mistrust or denial of a positive urine or blood pregnancy test result.^7,17,18^

A small number of studies have attempted to capture key aspects of pregnancy recognition beyond the binary question, “Are you currently pregnant?”. For example, recognition has been measured through survey questions on pregnancy testing,^18,19^ the repeated administering of pregnancy tests in longitudinal studies,^20^ and self-reports on timings of recognition.^4,19^ A small number of studies have incorporated information on delays to recognition. This includes open text responses for delays specifically related to taking a urine pregnancy test,^7^ checklists to understand if and why access to pregnancy-related care was delayed,^7^ questions about delayed recognition relating to menstruation and if a person was using contraception,^9,21^ and questions on pregnancy symptoms and “denial”.^10^

Informed by previous work on pregnancy recognition, the authors in this paper attempted to improve on existing approaches to capture this experience. This paper critically examines evidence from two studies that sought to improve on existing pregnancy recognition measures. Outcomes of both studies have been published previously^14,22–26^; this manuscript is a critical, comparative treatment of the pregnancy recognition variables in each study by the individuals who created those measures.

### AACSA and SMAN studies

This paper draws from two studies, which attempted to improve the capture of pregnancy recognition trajectories: the Adolescent Access to Contraception and Safe Abortion (AACSA) study conducted in Ethiopia, Malawi, and Zambia and a study on self-managed abortion in Nigeria (SMAN).

AACSA was a cross-sectional, mixed-methods study conducted in 2018–2019 with 313 adolescents aged 10–19 who sought abortion-related care in Ethiopia (*N* = 99), Malawi (*N* = 104), and Zambia (*N* = 110). The primary objective of the AACSA was to understand abortion and post-abortion trajectories among adolescents. Adolescents were invited to participate in the study if they presented at a study facility for an abortion or post-abortion care.

SMAN was a longitudinal study conducted in 2018 with 394 women aged 18–49 in Nigeria who completed a set of three surveys over a one-month period after buying misoprostol. The primary objective of the SMAN study was to understand women’s experiences using misoprostol obtained from drug sellers. Women were recruited through drug sellers if they were purchasing medication containing misoprostol.

The studies used different methods to capture pregnancy recognition. We examine the strengths and limitations of each method to collect data on pregnancy recognition trajectories, using descriptive statistics and authorial reflections. Both studies were conducted in urban areas. The AASCA study was conducted in facilities that provided abortions or post-abortion care in major cities in each of the three countries. The SMAN study purposively selected areas with post-secondary educational institutions under the assumption that these were populations more likely to self-manage using medication abortion.^24^

Ethical approval for the AACSA study was provided in Ethiopia (Ethiopian Public Health Institute: 154-2018), in Malawi (National Health Sciences Research Committee: 2023, 09/05/2018), in Zambia (ERES-2017-Nov-005), and in the UK (London School of Economics: 000606, 21/09/2017). Ethical approval for the SMAN study was provided by the National Health Research Ethics Committee in Nigeria (NHREC/01/01/2007), and the Institutional Review Board of Guttmacher Institute (IRB00002197, 29/11/2017) approved the study.

Informed consent was sought in both studies from a member of the research team. For minors who participated in the AACSA study, trained research assistants completed informed consent either from an accompanying parent or guardian (with the minor’s assent) or from the minor themselves.

### Survey questions

The two studies used different approaches to asking about pregnancy recognition (Table 1). In the AACSA study, two interviewers were used to collect mixed-method data on an adolescent’s abortion trajectory. One interviewer conducted a more conversational interview, captured through recording for qualitative analysis, while another interviewer completed a data sheet for quantitative analysis. All data were collected face to face, and both quantitative and qualitative data were captured in one interview to minimise the burden on an adolescent. The data sheet allowed for the collection of a single categorical response for how an adolescent “knew” they were pregnant, alongside open text for the second interviewer to add details on other factors that contributed to their pregnancy recognition.

**Table 1.** Data collection questions asked for each project

The AACSA project asked the following questions: – At the time you became pregnant with this pregnancy that has just ended, how did you know you were pregnant? [Mark X all mentioned] o Pregnancy test o Period late o Someone else noticed o Body changes o Anything else? Space was then given on the datasheet to collect any additional details of how an adolescent confirmed their pregnancy, which were then captured and added verbatim to the dataset for coding.
The SMAN study project asked the following questions on pregnancy awareness and confirmation: – For the pregnancy that you just tried to end [or period that returned], how did you discover you were pregnant? [TICK ALL THAT APPLY] o I intuitively felt pregnant o I experienced other symptoms of pregnancy (e.g. breast tenderness) o Menstrual period was late o A medical provider performed a bimanual exam o A medical provider drew my blood o A medical provider gave me a urine test o A medical provider performed an abdominal ultrasound o I went to a laboratory and they drew my blood o I went to a laboratory and they gave me a urine test o A drug seller sold me a urine test, which I self-administered o Other [please specify] – Did you take any tests to confirm you were pregnant? o No test o A medical provider performed a bimanual exam o A medical provider drew my blood o A medical provider gave me a urine test o A medical provider performed an abdominal ultrasound o I went to a laboratory and they drew my blood o I went to a laboratory and they gave me a urine test o A drug seller sold me a urine test, which I self-administered o Other [please specify] – Did you take any other tests to confirm you were pregnant? o No test o A medical provider performed a bimanual exam o A medical provider drew my blood o A medical provider gave me a urine test o A medical provider performed an abdominal ultrasound o I went to a laboratory and they drew my blood o I went to a laboratory and they gave me a urine test o A drug seller sold me a urine test, which I self-administered o Other [please specify]

The SMAN study included women who were recruited after informally purchasing misoprostol, screened for inclusion, and, if qualified, invited to participate in two interviews. The questionnaires were administered by telephone over approximately one month; pregnancy recognition was captured in the first interview. Response categories were not read out to women; relevant follow-up questions were asked based on previous responses.

### Reflexivity statement

Reflexivity requires engaging with gendered, racialised and other hierarchies within academia and research. These are embedded in historical and colonial contexts.^27^ Engaging with the growing critical work on reflexivity and positionality statements, we are aware of the limitations of our attempts to reflexively critique our work and hold ourselves accountable. Our thinking has been informed to date by the work of authors such as Gani and Khan on the role of positionality and reflexivity statements as functions of coloniality.^27^

This paper is a secondary data analysis of datasets from two research projects, one [SMAN] based in Nigeria and one a comparative study [AACSA] in Ethiopia, Malawi, and Zambia. The lead author [JS] was not involved in the conceptualisation or data collection processes of either. EC and TF co-led the AACSA project work, in collaboration with colleagues who have led papers published elsewhere. (see e.g.^22,28^) OO and AMM led and worked on the SMAN project, respectively, with collaborating organisations; OO is Nigerian and has lived, and has family who continue to live, in the context of the SMAN project, drawing on these experiences and her positionality through the course of the project. All authors are currently based at UK or US institutions with UK or US academic training in demography, anthropology, and public health. We are a mix of different academic and non-academic roles, including senior research roles [TF, OO, AMM] and academic staff [JS, EC].

The respondents who contributed the data to this article in the form of interviews and survey responses are women and girls who sought abortions in Ethiopia, Malawi, Nigeria, and Zambia. The data reflect what these women and girls wanted to tell researchers. Our interpretation and analysis in this paper only speak to a small component of these women and girls’ full experiences. Our paper seeks to consider how we may better capture this specific experience quantitatively, a method of evidence production that cannot be separated from its origins of making populations legible for the State.^29^ As authors, we are aware of the need to contend with our own roles in silencing aspects of the experiences of the women and girls who were interviewed. We hope, in this paper, that critically examining secondary data is a means to highlight the role of quantitative tools to capture experiences and flatten them, making it difficult to represent the voice, agency, and experiences of the women and girls the data claim to represent.

## Results

Adolescents aged 10–17 in the AASCA study reported that they knew they were pregnant due to menstrual changes, while a higher proportion of older adolescents aged 18–19 reported knowing through a positive medical [urine, blood, ultrasound] pregnancy test result. There were descriptive country-level differences: a lower proportion of adolescents in Malawi recognised that they were pregnant using medical pregnancy tests (31.7%, *n* = 33) than in Ethiopia (51.5%, *n* = 51) and Zambia (54.5%, *n* = 60).^14^^24^

The AACSA survey captured the complexities of adolescents’ pregnancy recognition trajectories through open text responses. Most adolescents in the AACSA study specified multiple factors that led to their pregnancy recognition; the majority cited between two and four recognition factors, with a range of one to seven factors. The highest proportion of adolescents (33.5%, n = 105) cited two factors. Most adolescents said that they “knew” they were pregnant after they used a medical pregnancy test (46.0%, *n* = 144), followed by recognition through late periods (41.2%, *n* = 129). A total of 201 (64.2%) adolescents took a test at some point in their trajectory, suggesting that a medical pregnancy test was not always the definitive factor in how they “knew” they were pregnant. Similarly, 216 of 313 (69.0%) adolescents reported late periods as a factor within their pregnancy recognition trajectory.

Nine adolescents identified bodily changes as the way they recognised they were pregnant in the closed question, but open text data captured through follow-up questions showed that bodily changes were mentioned 274 times across interviews. Factors included sickness (*n* = 58), breast tenderness or changes (*n* = 30), nausea (*n* = 29), abdominal pain (*n* = 28), and loss or changes in appetite (*n* = 22). In addition, adolescents mentioned headaches, weight change, fatigue, and bladder or bowel issues.

The role of other people in pregnancy recognition trajectories was identified in the closed responses. About 23 (7.3%) adolescents reported someone else noticing as the main reason they knew they were pregnant. Of these “other” people, seven were relatives (mothers, grandmothers, aunts), five were neighbours, and five were friends. Two were the sisters of friends/boyfriends, one was an employer, one a visitor to the household, and two were unspecified/general people around the adolescent. In open text responses, more adolescents (22.0%, *n* = 69) reported that someone else noticing was a factor in their pregnancy recognition trajectory but not the primary factor. The chronology of factors was not recorded.

Women in the SMAN study were first asked, “For the pregnancy that you just tried to end [or period that returned], how did you discover you were pregnant?”. No prompts were given, and interviewers recorded all factors that women mentioned. 43.4% (*n* = 171) of women gave two recognition factors leading them to recognise they were pregnant, with a range of between one to five recognition factors reported. The most frequently cited factor for respondents realising they were pregnant was a late menstrual period; 252 (64.0%) respondents cited this as at least one factor in their pregnancy discovery. About 160 (40.6%) bought a urine test from a drug seller to self-administer, 147 (37.3%) women reported pregnancy symptoms (not specified), while 107 (27.2%) reported that they intuitively knew they were pregnant. Of the women who responded “other” in response to how they discovered they were pregnant, three described taking a test, and three described bodily changes. One woman reported knowing she was pregnant from having had sex, explicitly linking the type and timing of intercourse she had to pregnancy risk.

In the SMAN study, a higher proportion (40.7%) of women aged 18–24 reported that they knew they were pregnant through a medical pregnancy test compared with other age groups, while among all age groups, having a late menstrual period was cited most frequently. The proportion of women who cited a medical pregnancy test as the way they recognised they were pregnant decreased with age; 35.1% for women aged 25–29, 32.4% for women aged 30–34, 30.5% for women aged 35–39, and 18.2% for women aged over 40.

Among SMAN respondents, 157 (39.8%) did not mention any testing in their initial responses to how they recognised they were pregnant. These women were subsequently asked follow-up questions about whether they did any tests for their pregnancy. This follow-up was asked after the initial, unprompted question about the factors that made them discover they were pregnant. About 90 (57.3%) of these women reported that they had done a test at some point, with 60 (38.2%) of those reporting that they did a self-administered urine test. Among the 237 women who reported taking a test, 41 (17.3%) said that they had done more than one test.

## Discussion

These two data collection experiences in sub-Saharan Africa highlight the complexities of capturing experiences of pregnancy recognition. Rather than a singular, confirmatory event, it was more common that adolescents and women cited multiple factors that led to them being aware of the pregnancy. Both approaches found that there was a heterogeneity in the methods that people used to recognise that they were pregnant. Menstrual changes and medical pregnancy testing were the most common definitive recognition factors reported; medical pregnancy testing was not always spontaneously mentioned in response to survey questions about pregnancy recognition.

The SMAN study provided insights into the kinds of medical pregnancy tests women used, and found some women took multiple medical pregnancy tests. For many respondents, medical pregnancy tests are just one piece of evidence that women gather during their pregnancy recognition process. The findings in this study emphasise the potential complications with existing tools that treat medical pregnancy testing as a singular measure to definitively determine when pregnancy confirmation occurred. Follow-up questions were important and suggested factors to include in the development of future categorical questions. The role of others in pregnancy recognition was an unexpected finding and should be considered for inclusion in future tool design. Moreover, the role of husbands/partners in pregnancy recognition should be explored.

In the AASCA study, results suggest that methods for recognising a pregnancy intersect with the structures and legal context. The lower proportion of adolescents in Malawi who used a pregnancy test might be related to the relatively restrictive legal environment of abortion in Malawi, with abortion heavily stigmatised and adolescents having less access to resources compared to Ethiopia and Zambia.^22^ The stigmatisation of abortion intersects with norms around adolescent sexuality,^30^ which may compound the need for adolescents in Malawi to maintain the secrecy of a possible pregnancy through the avoidance of buying pregnancy tests.

Recognition beyond medical pregnancy tests is critical. A late menstrual period is also worthy of further interrogation, as it may mean different things to different women. For example, we do not know what proportion of women might interpret a late period as a menstrual period that has not come on the expected day and so she begins to suspect she is pregnant the following day, as compared to what proportion of women wait until they have not menstruated for a full cycle (one or more) as a late period. Improved capturing of the pluralities of what is meant by “a late menstrual period” is critical information for understanding pregnancy recognition trajectories.

Moreover, more data to situate home urine tests in pregnancy recognition trajectories is essential, particularly with the increasing accessibility of self-managed medical abortions and the usefulness of tests to both confirm a pregnancy and a successful abortion.^31^ This includes data capturing the accessibility, availability, and preference for pregnancy tests. Future research should interrogate the relationship between adolescents' and women’s own knowledge and their attitudes and perceptions of tests as a form of confirmation. Adolescents' and women’s understandings of when in the pregnancy a test is more accurate, reasons they think self-administered urine tests may not be trustworthy, and preferred brands of tests are also worth further examination. Specific questions to respondents who reported they recognised a pregnancy due to a medical pregnancy test could clarify what led them to obtain a test in the first place. This would allow for including the role of others in possibly coercing or forcing medical pregnancy testing.

### Considerations

Neither tool was able to capture the ordering of factors nor the relative importance of different factors among adolescents and women coming to the recognition that they were pregnant in these particular settings. The data did not capture the costs of accessing and acquiring medical pregnancy tests; that path towards pregnancy recognition may be financially out of reach for some. Nor did it collect information on the pregnancy duration of respondents, as this is a complex estimation that was outside the remit of either study. The data examined are from four countries in Africa, and all come from adolescents and women seeking abortion. All four countries represent unique contexts; therefore, the patterns we identified among these respondents cannot be generalised. The two studies have different sampling frames and utilise different methods. Including the two different approaches allows for greater insights into pregnancy recognition but limits comparisons to consider the differences between contexts.

## Conclusion and recommendations

How people come to recognise they are pregnant can have significant implications for their ability to exercise their rights – for example, to antenatal care and to abortion-related care. Moreover, people have a right to recognise they are pregnant without interference from others, to make these decisions in autonomous ways that centre their choices. This descriptive analysis of two studies shows that pregnancy recognition can be a complex and multifaceted process. The dearth of data on pregnancy recognition limits the capacity to make evidence-based programmes and policies on improving the public’s awareness of pregnancy signs, as well as access to medical pregnancy tests for those who need them. Our critical reflections on these two case studies offer the following recommendations for designing questions to improve our understanding of pregnancy recognition trajectories:

1. Medical pregnancy testing should not be taken as the definitive factor that leads someone to recognise that they are pregnant. Surveys should include response categories for menstrual changes (with as much detail as feasible), physical symptoms of pregnancy, and other people noticing the pregnancy (including who noticed).
2. Quantitative health surveys, which are a critical component of SRH research and monitoring and evaluation, should consider what kind of medical pregnancy tests (if any) the respondent used, the timings of use, how many tests they took, and allow multiple responses to these questions. Follow-up questions about what prompted a medical pregnancy test should be asked, as well as if anyone else made them take the test (including interactions with healthcare providers and any costs). In settings where abortion is legally permitted, follow-up details on the timing and place of a test would offer insights on whether a test was done to receive an abortion. These nuanced data on pregnancy recognition may also be important for facility-based surveys with service users, as well as follow-up communication for pregnancy-related care, including abortion care.
3. Individuals who took multiple actions in the process of recognition should be given an opportunity to explain the value they place on these different actions, what they considered confirmatory and why. For example, which method of identification after multiple methods prompted them to take action, such as seek an abortion, confide in someone, seek antenatal care, etc. As we seek to better understand experiences, this could best be captured in open text responses until we have a better understanding of common perspectives.
4. Surveys would add greatly to our understanding of women’s experiences of pregnancy recognition if they were able to add questions on the duration of time between different components of a pregnancy recognition trajectory and reasons for the duration of time between different components.
5. Respondents should be asked to provide a ranking of the factors based on the importance they held for the respondent that led them to recognise a pregnancy, as this would potentially offer ways to understand primary and secondary factors of confirmation.
6. Further information on respondents’ perceived likelihood of getting pregnant at the time they became pregnant would allow for a greater understanding of health literacy as well as the role that perceived pregnancy risk plays in pregnancy recognition trajectories.
7. Capturing these trajectories in different geographic locations with different populations will help broaden our understanding of how these trajectories differ based on geographies and demographics.

Capturing pregnancy recognition trajectories will increase our understanding of key factors that shape the availability and accessibility of pregnancy-related care. This includes antenatal care pathways and care for at-risk women e.g. those who have experienced pregnancy loss, have chronic conditions, etc. As abortion restrictions on gestational age curtail abortion access, understanding who is most likely to have their access to abortion curtailed by later pregnancy recognition would help us better understand the impact of these laws.

## Author contributions

The paper concept was developed by JS and AM, with analysis by JS. Data curation and analysis of the SMAN project were conducted by JS, AM, and OO, while for the AASCA project, this was done by JS, EC, and TF. JS was responsible for writing the original draft, with input from all authors. All authors were involved in the review and editing of the draft.

## Data Availability

Data for the AACSA project are available on the UK Data Service: Coast, Ernestina (2024). *Improving Adolescent Access to Contraception and Safe Abortion in sub-Saharan Africa: Health System Pathways, 2017-2020*. [Data Collection]. Colchester, Essex: UK Data Service.10.5255/UKDA-SN-856965.

## Provenance statement

This article was not commissioned and went through external peer review.
